# 912. Influence of a Global Pandemic on Bronchiolitis at a Bronx Hospital

**DOI:** 10.1093/ofid/ofad500.957

**Published:** 2023-11-27

**Authors:** Michael Rosenberg, Karen Schaeffer, Alexandra J Boni, Christiaan Strydom

**Affiliations:** Jacobi Medical Center, Bronx, New York; NYC H+H Jacobi/ Albert Einstein College of Medicine, Bronx, New York; Jacobi Medical Center, Bronx, New York; Albert Einstein College of Medicine Jacobi Medical Center, New york, New York

## Abstract

**Background:**

Bronchiolitis is a common respiratory illness among young children, particularly during the winter season. The illness is usually caused by a viral infection, with the most common pathogens being respiratory syncytial virus (RSV), human rhinovirus (HRV), and human metapneumovirus (hMPV). The COVID-19 pandemic has led to changes in healthcare-seeking behavior and infection control practices that may have affected the incidence of other viral diseases.

**Methods:**

This study aimed to compare the rates of bronchiolitis at a community hospital in The Bronx, New York before, during, and after the COVID-19 pandemic and identify the viruses that caused it. We identified all patients admitted to the hospital with a diagnosis of bronchiolitis from July 2019 to January 2023. We conducted a retrospective analysis on these patients including length of hospital stay and viral test results.

**Results:**

We included a total of 220 bronchiolitis cases in the analysis. Prior to the COVID-19 pandemic the number of bronchiolitis admissions peaked from November 2019 through January 2020. During the first year of the pandemic, 2020, there was no significant bronchiolitis peak. In 2021, there was a longer season with bronchiolitis cases from July through January, although it did not have as sharp of a peak as 2019. In 2022, there was a large peak in September-December with almost double the cases at the peak compared to 2019. In almost half of the cases, RSV was identified. In one-fourth of cases, there was no organism identified. HRV and hMPV were the next most common viruses. Covid-19 identified as a cause of bronchiolitis in October and November 2022 only.

Bronchiolitis Admissions by Month July 2019 to January 2023

**Figure:**
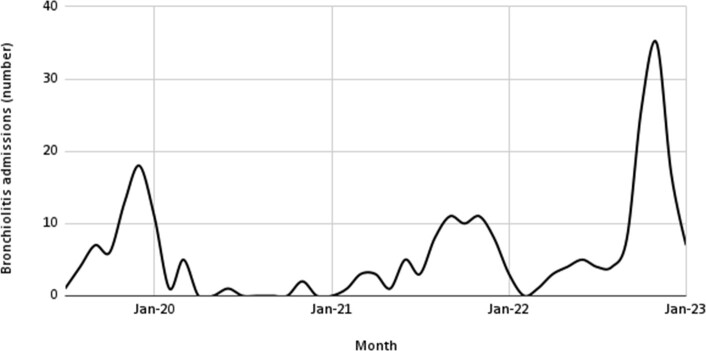

**Conclusion:**

Our findings show a significant decrease in the rate of bronchiolitis during the COVID-19 pandemic compared to the pre-pandemic period, with an increased rate of bronchiolitis after the COVID-19 pandemic. These findings suggest that the implementation of public health measures to mitigate the spread of COVID-19 may have inadvertently reduced the transmission of other respiratory viruses, with a shift in epidemiology after these measures were lifted. However, ongoing surveillance is needed to determine whether this shift in viral epidemiology is temporary or represents a long-term trend.

**Disclosures:**

**All Authors**: No reported disclosures

